# Biphasic synovial sarcoma in the cervical spine: Case report

**DOI:** 10.1186/2045-709X-19-12

**Published:** 2011-05-23

**Authors:** Stephen M Foreman, Michael J Stahl

**Affiliations:** 1 Private practice of chiropractic, West Hills, California, USA

## Abstract

Synovial sarcoma is a rare malignant neoplasm of soft tissue that typically arising near large joints of the upper and lower extremities in young adult males. Only 3% of these neoplasms have been found to arise in the head and neck region. To our knowledge, there are limited reports in the literature of this neoplasm in the cervical spine.

A case of biphasic synovial sarcoma of the cervical spine is reviewed. A 29 year-old male presented with pain on the left side of the cervical spine. Physical examination revealed a global loss of cervical motion and large, palpable mass in the left paravertebral area. The long-delayed Magnetic Resonance (MR) scan revealed a soft tissue mass measuring 8.3 centimeters (cm) × 5.7 cm that was surgically removed. A malignant biphasic synovial sarcoma was diagnosed on pathologic examination.

The clinical and imaging findings of an atypically located synovial sarcoma are reviewed. This case report emphasizes the consequences of a limited differential diagnosis, prolonged treatment and the failure to perform timely diagnostic imaging in the presence of a paraspinal mass.

## Background

Synovial sarcoma is a seldom encountered, aggressive malignant neoplasm of soft tissue that typically arises near large joints of the upper and lower extremities in young adults. Synovial sarcomas account for 7-10% of all soft-tissue sarcomas [1]. The anatomical distribution of synovial sarcomas is well documented with 85% located in the extremities [2] and just 3% located in the head and neck region [3]. Fang, et al [4] confirmed the low incidence of synovial sarcoma in the spine in their review of 191 cases and the anatomical distribution of these tumors is seen in Table 1. The designation of the "head and neck" location is somewhat misleading, as it does not usually indicate involvement in the spine. The preponderance of cases with the "head and neck" designation are located in the hypopharynx [5] and few synovial sarcomas are located in the cervical or other regions of the spine.

**Table 1:** Anatomical distribution of 191 cases of synovial sarcoma

Location	Number of cases	Percentage
Lower limbs or buttocks	98	51.3%
Upper limbs or shoulders	39	20.4%
Pelvis	19	9.9%
Chest	13	6.8%
Abdominal wall	12	6.7%
Head and neck	6	3.1%
Trunk	4	2%

Adapted from Fang Z, Chen J, Teng S, Chen Y, Xue R: **Analysis of soft tissue sarcomas in 1118 cases**. *Chin Med J *2009, **122**(1):51-53 [4].

This paper describes the clinical, radiological and pathological findings of a synovial sarcoma that was located in the lower cervical paravertebral space. Although the radiological and clinical features of a typically located synovial sarcoma are documented in the literature, our review of the literature reveals limited reports of synovial sarcoma arising from the cervical spine [6].

## Case presentation

A 29 year-old male presented with muscle discomfort and pain in the posterior left cervical spine, especially after weight lifting. There was no history of recent trauma. Five years prior to presentation, the patient had sustained cervical injuries from a motor vehicle accident. The patient's motor vehicle related cervical spine complaints resolved with manipulation and physical therapy shortly after the accident. The patient recently returned to care and a regional orthopedic and neurological examination was performed with findings of "myofascial trigger points on the left levator scapulae muscle". The patient was diagnosed with "myofascial pain syndrome of the left levator scapulae" and was placed on a course of care that consisted of manipulation, post-isometric relaxation, stretching and "post-facilitated stretching once the trigger points are resolved." No initial imaging studies were performed. The presenting size of the paravertebral "trigger point" was not documented in the record.

The patient underwent a course of chiropractic care, which totaled 24 treatments over a 13-month period. The clinical record noted both positive and negative subjective responses to the conservative care. Multiple comments in the chart notes indicated the "trigger point" and "swelling" were worsening during the course of care, but these observations of an enlarging mass were not accompanied by any change in treatment, re-evaluation or additional investigation with any form of diagnostic imaging. The patient eventually discontinued care as the paravertebral mass had steadily grown and was now clearly visible on visual inspection of the area. The subsequent treating clinician ordered a MR scan.

### Imaging findings

**Figure 1:**
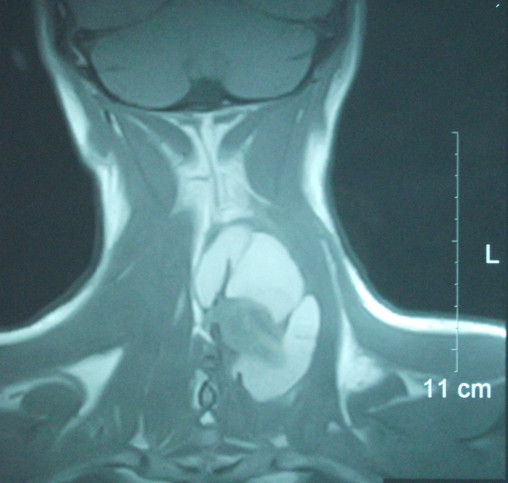
**Coronal MR of tumor extending from C3-T2**. MR scan, T1 weighted, coronal view without contrast, reveals a kidney bean shaped cystic mass that extends from C3 to T1. The adjacent 11 cm measurement scale was used to determine this mass measured approximately 8.3 cm × 5.7 cm.

**Figure 2:**
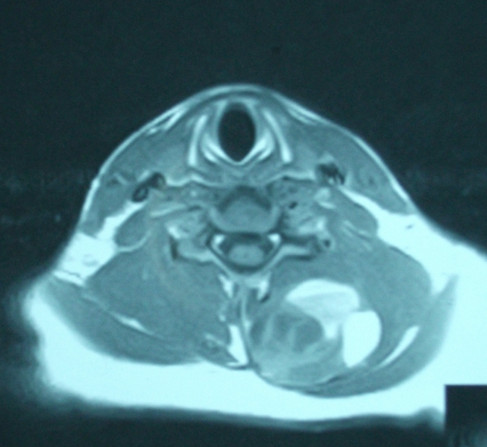
**Axial MR scan of cystic tumor**. This T1 weighted MR scan, axial view of the tumor, reveals the cystic nature of the lesion with central low signal tumor components and higher signal peripheral proteinaceous/hemorrhagic components. The mass does abut the posterior elements but there is no sign of communication with the central canal.

**Figure 3:**
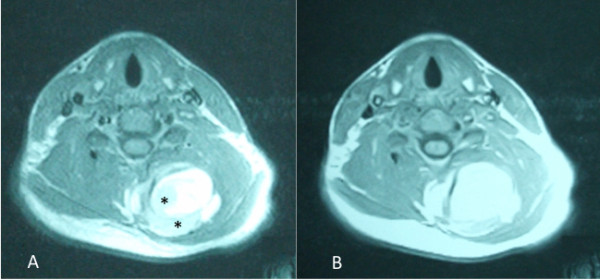
**Pre and Post Gadolinium Axial MR scans**. A non-enhanced axial T1 weighted image (3A) reveals multiple levels of signal intensity. The area of lower signal intensity (asterisks) represents the tumor and the higher signal is consistent with hemorrhage and fibrous tissue. 3B reflects increased signal intensity in the tumor after administration of Gadolinium.

MR images with pre and post gadolinium axial and coronal T1 weighted images revealed a complicated mass extending from the C3-C4 level to the T1-T2 level. The coronal MR revealed a septated kidney bean shaped mass with a large portion demonstrating elevated signal intensity on T1 weighted images (Figure 1). On MR imaging, synovial sarcomas usually appear as a heterogeneous soft-tissue mass and may have a multilocular appearance. The multiple signal intensities of synovial sarcomas on non-enhanced studies are the result of solid and cystic components with hemorrhage and fibrous tissue [7]. Non-enhanced axial T1 weighted images noted the tumor abutted the posterior elements but there was no communication with the central canal (Figure 2). Pre and post gadolinium axial T1 weighted images demonstrated increased enhancement of the tumor nidus (Figure 3). The overall measurements of this tumor mass were 8.3 cm × 5.7 cm × 3.7 cm. Initial impressions were consistent with neoplasm and a sarcoma could not be excluded. Computerized tomography (CT) of the neck was recommended to help differentiate the tumor.

**Figure 4:**
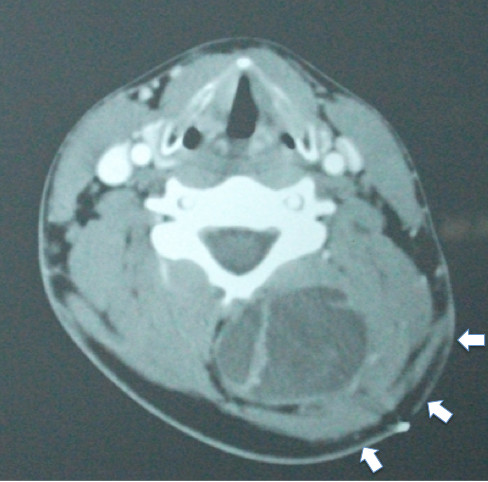
**Axial CT scan of the cervical spine**. 3 mm transaxial CT scan with nonionic intravenous contrast. The non-enhancing cystic mass involves the paraspinal musculature and is adjacent to the posterior elements particularly the spinous process and laminae of C4. Note the prominent distortion of the soft tissues overlying the tumor (white arrows) when compared to the unaffected side.

CT scan images were obtained following nonionic intravenous contrast and compared to the earlier MR study. The axial CT revealed a non-enhancing cystic mass adjacent to the posterior elements at C4 and C5 (Figure 4). The list of differential considerations at the time included epidermoid, hemangio-pericytoma, lymphangioma and sarcoma.

### Pathological findings

The resected tumor was evaluated via frozen section and revealed a moderately cellular cystic/intracystic neoplasm composed of two morphologically different tumor cell types (biphasic). One cell type was spindle and the other was epithelial. The spindle cells were similar to fibrosarcoma. The epithelial cells presented as focal glandular formations with clusters and trebeculae. Calcification and ossification, often seen on CT imaging of these tumors, were noted in the pathological study but were never visualized in the imaging studies. The final pathological diagnosis was "synovial sarcoma, biphasic type, intracystic."

### Treatment

The prevailing therapeutic approach to high-grade soft tissue sarcomas is wide surgical resection followed by radiation, chemotherapy or both [7]. The patient in this case report underwent subtotal resection due to the size of the tumor. Seven weeks of post-operative radiation therapy was received and this was followed by a course of chemotherapy. This patient is now six years post resection without recurrence of the tumor.

## Discussion

Synovial sarcoma is a malignant neoplasm of soft tissue that typically arises near large joints of the upper and lower extremities in the young adult male, particularly the knee; however, they do not arise from synovial tissue [1,8,9] but from malignant degeneration of primitive mesenchymal cells [9]. The microscopic appearance of the degenerated mesenchymal cells is remarkably similar to synovial tissue, hence the name of the tumor.

Presenting clinical symptoms vary according to the size and location of the tumor. Those tumors arising in an extremity may present initially with swelling, pain or tenderness. Limitation in motion may be noted if the tumor is located near a joint. The non-specific nature of the symptoms may initially be interpreted as more commonly encountered soft tissue entities such as bursitis and myositis. The increasing size of the tumor also has the ability to compress nerves and result in the gradual onset of neurological deficits. A high degree of clinical suspicion, along with the observation of gradually developing mass should prompt the use of diagnostic imaging even in the absence of a history of trauma.

Synovial sarcoma of the spine is quite uncommon and early diagnosis may be difficult without advanced imaging. The tumor may cause a variety of symptoms, again depending on the size and location of the mass. Neurological compromise is also possible with tumors located near the spine. A case of paravertebral synovial sarcoma in the lumbar spine was noted to produce a grade III weakness in dorsiflexion in the right great toe and decreased sensation of the L4-5 dermatome [10]. A palpable cervical or pharyngeal mass, often with localized pain may signal the presence of the tumor [1]. Those patients with pharyngeal tumors may also present with symptoms such as dysphagia, hoarseness or dyspnea.

Synovial sarcoma occurs in 2 histological subtypes: the biphasic form contains elements of both epithelial and spindle cells and the monophasic type contain only spindle cells [10].

Synovial sarcomas may aggressively grow and imaging studies have shown they vary in size between 2 and 9 cm [11,12]. Detection of the tumor at a smaller size is believed to affect long term prognosis, which was found to be better in patients whose tumors were ≤ 4 cm [5].

Synovial sarcomas are usually treated aggressively with wide excision with negative margins, often including removal of adjacent muscle groups and even total amputation [1,13]. Limited excision is unfortunately associated with a high incidence of local recurrence (60-90%) within 2 years of the original surgery [14]. The surgical excision is followed by post-operative radiotherapy and chemotherapy to help control metastasis [15,16].

## Conclusions

This case demonstrates the ever-present potential for an uncommon condition to present in an atypical location in the ambulatory outpatient setting. This patient would have benefitted from earlier diagnostic imaging and consultation with other practitioners when the patient began to develop a paraspinal mass. Although rare, synovial sarcomas and other forms of soft tissue tumor should be included in the differential diagnosis of paraspinal masses in patients, irrespective of their response to conservative care.

## Consent

Written informed consent was obtained from the patient for publication of this Case report and any accompanying images. A copy of the written consent is available for review by the Editor-in-Chief of this journal.

## Competing interests

The authors declare they have no competing interests. SMF was involved in this case as a consultant for the patient after the tumor had been resected.

## Authors' contributions

SMF conducted the initial review of the case and prepared the first draft of the manuscript. MJS participated in the conception of the report, the revision and coordination of the final manuscript. Both authors read and approved of the final manuscript.
